# First Request First Service Entanglement Routing Scheme for Quantum Networks

**DOI:** 10.3390/e24101404

**Published:** 2022-10-01

**Authors:** Si-Chen Li, Bang-Ying Tang, Han Zhou, Hui-Cun Yu, Bo Liu, Wan-Rong Yu, Bo Liu

**Affiliations:** 1 College of Computer, National University of Defense Technology, Changsha 410073, China; 2 Information and Navigation College, Air Force Engineering University, Xi’an 710077, China; 3 College of Advanced Interdisciplinary Studies, National University of Defense Technology, Changsha 410073, China

**Keywords:** quantum network, entanglement routing, active wavelength multiplexing

## Abstract

Quantum networks enable many applications beyond the reach of classical networks by supporting the establishment of long-distance entanglement connections, and are already stepped into the entanglement distribution network stage. The entanglement routing with active wavelength multiplexing schemes is urgently required for satisfying the dynamic connection demands of paired users in large-scale quantum networks. In this article, the entanglement distribution network is modeled into a directed graph, where the internal connection loss among all ports within a node is considered for each supported wavelength channel, which is quite different to classical network graphs. Afterwards, we propose a novel first request first service (FRFS) entanglement routing scheme, which performs the modified Dijkstra algorithm to find out the lowest loss path from the entangled photon source to each paired user in order. Evaluation results show that the proposed FRFS entanglement routing scheme can be applied to large-scale and dynamic topology quantum networks.

## 1. Introduction

Quantum networks are supported for creating, transmitting, and processing quantum information [1,2,3]. The vital function of quantum networks is to establish remote entanglement connections, which supports plenty of important applications beyond the reach of classical networks [4,5], such as highly secure communication [6,7,8,9,10,11], distributed quantum computing [12,13,14], remote quantum clock synchronization [15,16,17], and distributed quantum sensing [18,19,20].

So far, amounts of trusted relayed quantum networks have been successfully demonstrated, such as the DARPA quantum network [21,22], the SECOQC QKD network [23], the Tokyo QKD network [24], and the integrated space-to-ground quantum network over 4600 kilometers [25]. Although trusted relayed quantum networks form a primary stepping stone toward a quantum internet, the end-to-end transmission of qubits is not allowed [26]. On the other hand, constructing the quantum memory-assisted network still faces lots of challenges, mainly limited by the overall storage and retrieval efficiency [27,28,29,30]. The current status of quantum networks is already stepped into the entanglement distribution network stage.

Establishing the entanglement connections has been a key challenge for large-scale and long-distance quantum networks. To address this, a fully connected entanglement network [31] was first implemented with the dense wavelength division multiplexing (DWDM) strategy, but $O(n^{2})$ wavelength channels are required for *n* users, which limits the network scale to a few users [32,33,34]. Afterward, a fully connected quantum network on a city-wide scale with passive beam splitters and DWDMs was demonstrated in 2020, which decreased the requirement of the wavelength channels to O(n) [32]. Recently, reconfigurable and flexible entanglement networks were established with wavelength selective switches (WSS), where adaptive bandwidth management was performed to satisfy the dynamic connection demands of distant users [35,36,37,38,39,40,41]. Therefore, the entanglement routing with active wavelength multiplexing schemes is desiderated for constructing entanglement distribution networks with various topologies [42,43,44,45]. In the meantime, existing quantum routing schemes [2,46,47,48,49,50,51,52] are mainly focused on trusted node networks and quantum memory-assisted networks, and cannot be applied to quantum entanglement distribution networks, where time division multiplexing (TDM) and wavelength division multiplexing (WDM) techniques need to be adopted to forward entangled photons.

In this article, the entanglement distribution network is abstracted into a directed graph firstly, where the nodes represent the entangled photon source, the users and the wavelength routing devices, the edges stand for the quantum links among nodes. Especially, the pass-through loss matrix among all ports within a node is defined for each supported wavelength channel. Afterwards, we propose a novel first request first service (FRFS) entanglement routing scheme for quantum networks, which performs the modified Dijkstra algorithm to find the lowest loss path from the entangled photon source to each paired users in order. Finally, the evaluation results show that the proposed FRFS entanglement routing scheme can be applied to the large-scale and dynamic topology quantum networks.

## 2. Preliminaries

In order to evaluate the link performance between arbitrary paired communication users (Alice and Bob), a brief introduction about the bi-photon entanglement distribution knowledge is given in this section. Here, we assume that the polarized continuous-wave pumped entangled photon source is performed for Alice and Bob. The overall efficiency between the source and Alice (Bob) is $\eta_{A}$ ($\eta_{B}$). The brightness of the photon source is *B*. Thus, the measured single photon counts for Alice and Bob can be calculated by [53]

(1) $$S_{A}^{m}=B\eta_{A}+{\mathrm{DC}}_{A}\;\mathrm{and}\;S_{B}^{m}=B\eta_{B}+{\mathrm{DC}}_{B},$$

where ${\mathrm{DC}}_{A}$ and ${\mathrm{DC}}_{B}$ are the dark counts.

The total measured coincidences count between Alice and Bob ${\mathrm{CC}}^{m}$ is defined as

(2) $${\mathrm{CC}}^{m}=\eta^{t_{\mathrm{CC}}}{\mathrm{CC}}^{t}+{\mathrm{CC}}^{\mathrm{acc}},$$

where $t_{\mathrm{CC}}$ is the coincidence window, $\eta^{t_{\mathrm{CC}}}$ is the coincidence-window dependent detection efficiency. ${\mathrm{CC}}^{t}$ is the true coincident counts, which represents the rate of the events that two photons of a pair must be detected to observe their polarization correlation. ${\mathrm{CC}}^{\mathrm{acc}}$ is the accidental coincidence count.

The true coincidence count rate is calculated as

(3) $${\mathrm{CC}}^{t}=B\eta_{A}\eta_{B}.$$

The count rate of accidental coincidences in window $t_{\mathrm{cc}}$ is given by

(4) $${\mathrm{CC}}^{\mathrm{acc}}=S_{A}^{m}S_{B}^{m}t_{\mathrm{CC}}.$$

The count rate of the erroneous coincidences ${\mathrm{CC}}^{\mathrm{err}}$ is calculated as

(5) $${\mathrm{CC}}^{\mathrm{err}}=\eta^{t_{\mathrm{CC}}}{\mathrm{CC}}^{t}e^{\mathrm{pol}}+\frac{1}{2}{\mathrm{CC}}^{\mathrm{acc}},$$

where $e^{\mathrm{pol}}$ is the system polarization error probability.

Therefore, the quantum bit error rate (QBER ER) can be expressed by

(6) $$ER=\frac{{\mathrm{CC}}^{\mathrm{err}}}{{\mathrm{CC}}^{m}}=\frac{\eta^{t_{\mathrm{CC}}}{\mathrm{CC}}^{t}e^{\mathrm{pol}}+\frac{1}{2}{\mathrm{CC}}^{\mathrm{acc}}}{\eta^{t_{\mathrm{CC}}}{\mathrm{CC}}^{t}+{\mathrm{CC}}^{\mathrm{acc}}}.$$

In this paper, we assume that Alice and Bob perform the BBM92 protocol to generate the information-theoretical-secure keys. Since the noise parameters are independent of measurement settings, we assume that the phase error equals to ER. Thus, the secure key rate ($R^{s}$) can be expressed as [54]

(7) $$R^{s}=\frac{1}{2}{\mathrm{CC}}^{m}\left[1-f\left(ER\right)H_{2}\left(ER\right)-H_{2}\left(ER\right)\right],$$

where f(ER) is the information reconciliation efficiency and $H_{2}\left(x\right)$ is the binary Shannon entropy, which is defined as:

(8) $$H_{2}\left(x\right)=-x\log_{2}\left(x\right)-\left(1-x\right)\log_{2}\left(1-x\right).$$

## 3. Modeling for Entanglement Distribution Network

### 3.1. Network Components

**Figure 1:**
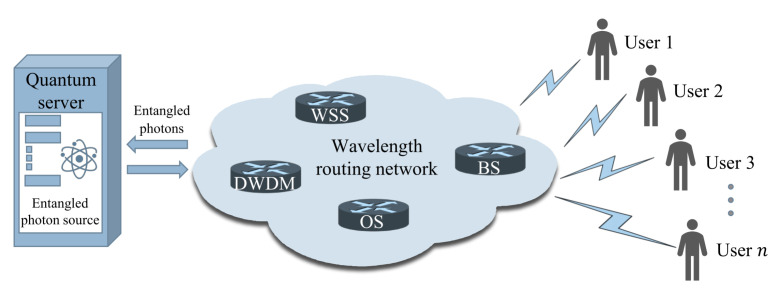
Schematic diagram of the entanglement distribution network. The entangled photon source and WSS are located at the quantum server to provide entangled photons. The entangled photons are transmitted to the users through the wavelength routing network, which is mainly connected via DWDM, BS, OS and, WSS devices. Then the users establish communication with others by accessing the switching network. DWDM: Dense Wavelength Division Multiplexer, BS: Beam Splitter, OS: Optical Switch, WSS: Wavelength Selective Switch.

As shown in Figure 1, the components of the entanglement distribution network can be abstracted into three types: Quantum server, wavelength routing network, and users.

- Quantum server. A quantum server provides and controls the wavelength switching of entangled photons.
- Wavelength routing network. A wavelength routing network is responsible for dividing the entangled bandwidth into multiple wavelength channels and routing them to distant users with OS, WSS, and other wavelength switching devices.
- Users. Users perform various protocols to generate secure keys, synchronize their clocks and so on.

The entanglement distribution network can be described as a graph G=〈V,E〉, where the node set *V* consists of the quantum server, wavelength routing devices, and users, and the edge set *E* includes the fiber or free-space links among the nodes.

### 3.2. Modeling of Nodes

*V* is the set of *k* nodes, each node $v_{i}$ is a bi-variate vector, defined as

(9) $$v_{i}≜\left(W_{i},T_{i}\right),$$

where 1≤i≤k. $T_{i}$ is the supported wavelength channel vector, consisting of the channel indexes defined by the International Telecommunication Union (ITU) Grid C-Band (100 GHz Spacing) standard. The size of $T_{i}$ shows the divided number of supported wavelength channels of $v_{i}$. For different type of $v_{i}$ considered in this article, $T_{i}$ is defined as

(10) $$T_{i}=\left\{\begin{array}{cc} {}[-3], & \mathrm{Entanglement}\;\mathrm{source}\\ {}[-2], & \mathrm{User}\\ {}[-1], & \mathrm{BS}\\ {}[0], & \mathrm{OS}\;\mathrm{or}\;\mathrm{AOS}\\ \left[{\mathrm{ch}}_{1},{\mathrm{ch}}_{2},\cdots ,{\mathrm{ch}}_{|T_{i}|}\right], & \mathrm{DWDM}\;\mathrm{or}\;\mathrm{WSS} \end{array}\right..$$

For the nodes implemented with entangled photon source, user, BS, and time-division multiplexing devices (such as OS and AOS), $|T_{i}|=1$, which means all the wavelengths can be routed through the node $v_{i}$. For the nodes implemented with DWDM and WSS ($|T_{i}|\geq 2$), the allowed wavelength channel index is varying from 1 to 72.

Assume that $m_{i}$ is the connection ports number of $v_{i}$. $W_{i}$ is defined as the pass-through loss matrix vector, consisting of $|T_{i}|$ square matrices, shown as

(11) $$W_{i}=\left[W_{i}^{1},W_{i}^{2},\cdots ,W_{i}^{|T_{i}|}\right],$$

where $W_{i}^{j}$ is the pass through matrix of *j*-th wavelength channel of $v_{i}$, defined as

(12) $$W_{i}^{j}=\left[\begin{array}{ccc} w_{11}^{ij} & \cdots & w_{1m_{i}}^{ij}\\ \vdots & \ddots & \vdots \\ w_{m_{i}1}^{ij} & \cdots & w_{m_{i}m_{i}}^{ij} \end{array}\right],$$

where $1\leq j\leq |T_{i}|$. Each element $w_{\alpha \beta }^{ij}$ is the insertion loss from port α to port β of the *j*-th wavelength channel for node $v_{i}$, $1\leq \alpha ,\beta \leq m_{i}$. If port β cannot be reached from port α, $w_{\alpha \beta }^{ij}=\infty$.

### 3.3. Modeling of Edges

Assume $e_{\alpha \beta }^{ij}$ is the edge (fiber or free-space link) from the α-th port of node $v_{i}$ to the β-th port of node $v_{j}$, where 1≤i,j≤k, $1\leq \alpha \leq m_{i}$ and $1\leq \beta \leq m_{j}$. $e_{\alpha \beta }^{ij}$ is a five-variable vector defined as

(13) $$e_{\alpha \beta }^{ij}=\left(i,\alpha ,j,\beta ,c_{\alpha \beta }^{ij}\right),$$

where $c_{\alpha \beta }^{ij}$ is the link loss and $e_{\alpha \beta }^{ij}\left[\gamma \right]$ represents the γ-th element in $e_{\alpha \beta }^{ij}$, $\gamma \in \left\{1,2,3,4,5\right\}$. If there is no link between the α-th port of node $v_{i}$ and the β-th port of node $v_{j}$, $c_{\alpha \beta }^{ij}=\infty$.

Thus, the link loss matrix from node $v_{i}$ to node $v_{j}$ can be defined as

(14) $$C_{ij}=\left[\begin{array}{ccc} c_{11}^{ij} & \cdots & c_{1m_{j}}^{ij}\\ \vdots & \ddots & \vdots \\ c_{m_{i}1}^{ij} & \cdots & c_{m_{i}m_{j}}^{ij} \end{array}\right].$$

### 3.4. Instantiation

**Figure 2:**
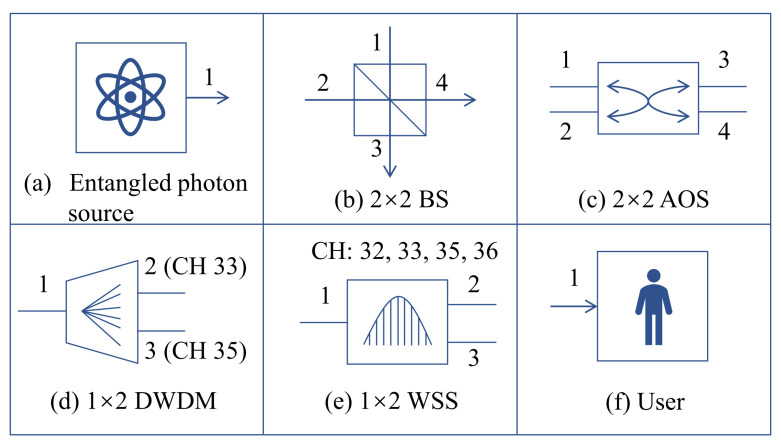
Schematic diagram of commonly implemented nodes. (**a**) Entangled photon source. (**b**) 2×2 beam splitter (BS). (**c**) 2×2 all-pass optical switch (AOS). (**d**) 1×2 dense wavelength division multiplexer (DWDM), CH33 and CH35 are supported here. (**e**) 1×2 wavelength selective switch (WSS), CH32, CH33, CH35 and CH36 are supported here. (**f**) User.

Here, we give the specific instantiations of common implemented nodes, including entangled photon source, 2×2 BS, 2×2 AOS, 1×2 DWDM, 1×2 WSS and user, shown in Figure 2.

The entangled photon source S can be instantiated as

(15) $$v_{S}=\left(\left[\begin{array}{c} \infty \end{array}\right],\left[-3\right]\right),$$

where the entangled photon source has 1 output port.

The 2×2 BS can be instantiated as

(16) $$v_{\mathrm{BS}}=\left(\left[\begin{array}{cccc} \infty & \infty & 3 & 3\\ \infty & \infty & 3 & 3\\ 3 & 3 & \infty & \infty \\ 3 & 3 & \infty & \infty \end{array}\right],\left[-1\right]\right),$$

where the pass-through loss among the BS ports is assumed to 3 dB.

The 2×2 AOS can be instantiated as

(17) $$v_{\mathrm{AOS}}=\left(\left[\begin{array}{cccc} \infty & 1 & 1 & 1\\ 1 & \infty & 1 & 1\\ 1 & 1 & \infty & 1\\ 1 & 1 & 1 & \infty \end{array}\right],\left[0\right]\right),$$

where the pass-through loss among the AOS ports is assumed to 1 dB.

The instantiation of the 1×2 DWDM is shown as

(18) $$v_{\mathrm{DWDM}}=\left(\left[\left[\begin{array}{ccc} \infty & 0.5 & \infty \\ 0.5 & \infty & \infty \\ \infty & \infty & \infty \end{array}\right],\left[\begin{array}{ccc} \infty & \infty & 0.5\\ \infty & \infty & \infty \\ 0.5 & \infty & \infty \end{array}\right]\right],\left[33,35\right]\right),$$

where the pass-through loss among the DWDM ports is assumed to 0.5 dB and 2 wavelength channels are supported by the DWDM.

The instantiation of the 1×2 WSS is shown as

(19) $$v_{\mathrm{WSS}}=\left(W_{\mathrm{WSS}},T_{\mathrm{WSS}}\right),$$

where

(20) $$W_{\mathrm{WSS}}=\left[\left[\begin{array}{ccc} \infty & 5 & 5\\ 5 & \infty & 5\\ 5 & 5 & \infty \end{array}\right],\cdots ,\left[\begin{array}{ccc} \infty & 5 & 5\\ 5 & \infty & 5\\ 5 & 5 & \infty \end{array}\right]\right]$$

and

(21) $$T_{\mathrm{WSS}}=\left[32,33,35,36\right].$$

Here, the pass-through loss among the WSS ports is assumed to 5 dB and 4 wavelength channels are supported by the WSS.

The user can be instantiated as

(22) $$v_{\mathrm{User}}=\left(\left[\begin{array}{c} \infty \end{array}\right],\left[-2\right]\right),$$

where the user has 1 receive port.

**Figure 3:**
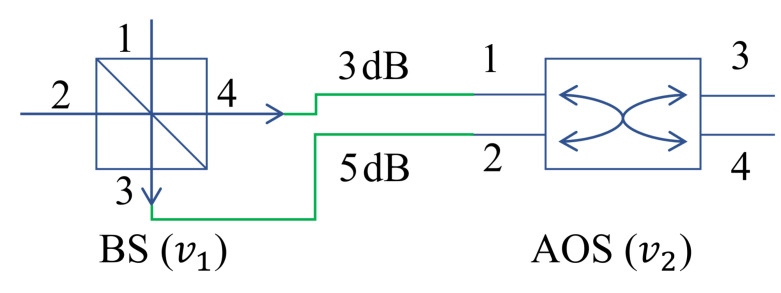
Schematic diagram of edges between a 2×2 BS and 2×2 AOS. The edges between BS and AOS are indicated by green lines.

As shown in Figure 3, we take the links between a 2×2 BS ($v_{1}$) and 2×2 AOS ($v_{2}$) as the examples to explain the modeling of edges.

The edges between $v_{1}$ and $v_{2}$ are instantiated as

(23) $$\begin{array}{c} e_{4,1}^{1,2}=\left(1,4,2,1,3\right), \end{array}$$

(24) $$\begin{array}{c} e_{3,2}^{1,2}=\left(1,3,2,2,5\right), \end{array}$$

(25) $$\begin{array}{c} e_{1,4}^{2,1}=\left(2,1,1,4,3\right), \end{array}$$

(26) $$\begin{array}{c} e_{2,3}^{2,1}=\left(2,2,1,3,5\right). \end{array}$$

The link loss matrix for connecting $v_{1}$ and $v_{2}$ can be described as

(27) $$C_{1,2}=\left[\begin{array}{cccc} \infty & \infty & \infty & \infty \\ \infty & \infty & \infty & \infty \\ \infty & 5 & \infty & \infty \\ 3 & \infty & \infty & \infty \end{array}\right].$$

(28) $$C_{2,1}=\left[\begin{array}{cccc} \infty & \infty & \infty & 3\\ \infty & \infty & 5 & \infty \\ \infty & \infty & \infty & \infty \\ \infty & \infty & \infty & \infty \end{array}\right].$$

## 4. First Request First Service Entanglement Routing Scheme

### 4.1. The Proposed FRFS Scheme

Assume that the entanglement distribution network is G=〈V,E〉, which consists of *k* nodes. For given arbitrary paired distant users (with node index of *i* and *j*), an efficient entanglement routing scheme is required to find a path through the entangled photon source in the large-scale network with dynamic topologies. The brightness *B* of the entangled photon source, $\eta^{t_{\mathrm{CC}}}$, $e^{\mathrm{pol}}$, ${\mathrm{DC}}_{i}$, ${\mathrm{DC}}_{j}$ and $t_{\mathrm{CC}}$ of users can be treated as constant parameters once the network *G* is constructed.

The coincidence counts ${\mathrm{CC}}^{m}$ and other evaluation criteria of the entanglement distribution between node *i* and node *j*, is usually negatively correlated with $l_{i}$ and $l_{j}$, where $l_{i}$ ($l_{j}$) is the link loss from the entangled photon source to node *i* (node *j*). Meanwhile, the network often faces the problem of limited path resources when assigning the path with the best evaluation criteria to multiple users requesting resources. Therefore, we propose a novel first request first service (FRFS) routing scheme for the entanglement distribution network with active wavelength multiplexing strategies, which finds the lowest loss path from an entangled photon source to each user in order.

Assume that the index of entangled photon source is *s*, the paired wavelength channels of signal and ideal photons for node *i* and *j* are ${\mathrm{ch}}_{i}$ and ${\mathrm{ch}}_{j}$. $p_{i}$ ($p_{j}$) is defined as the path between node *s* to node *i* (*j*). The link loss matrix for the network *G* can be derived from *V* and *E*. Thus, our proposed FRFS entanglement routing scheme mainly includes four procedures.

First of all, for the node *i* and the wavelength channel ${\mathrm{ch}}_{i}$, calculate $p_{i}$ with the modified Dijkstra algorithm in the network *G*, which is described in Section 4.2.

Secondly, for the wavelength channel ${\mathrm{ch}}_{i}$, the correlated import and output ports of all edges in $p_{i}$ are marked as the in use status by the “Lock” operation, defined as $V=Lock(V,p_{i},{\mathrm{ch}}_{i})$.

Thirdly, for the node *j* and the wavelength channel ${\mathrm{ch}}_{j}$, calculate $p_{j}$ with the modified Dijkstra algorithm in the updated network *G*.

Finally, for the wavelength channel ${\mathrm{ch}}_{j}$, the correlated import and output ports of all edges in $p_{j}$ are marked as the in use status, by the operation $V=Lock(V,p_{j},{\mathrm{ch}}_{j})$.

Once the entanglement distribution request is released by node *i* and node *j*, conduct the $V=Unlock(V,p_{i},{\mathrm{ch}}_{i})$ and $V=Unlock(V,p_{j},{\mathrm{ch}}_{j})$ to release the entanglement network link resources consumed by path $p_{i}$ and $p_{j}$.

**Definition** **1.** *$M=GM(v_{x},{\mathrm{ch}}_{x})$. Getting the pass-through loss matrix of node v for wavelength channel ${\mathrm{ch}}_{x}$ as*

(29) $$M=\left\{\begin{array}{cc} W_{x}^{1} & |T_{x}|=1\\ W_{x}^{{\mathrm{ch}}_{x}} & {\mathrm{ch}}_{x}\in T_{x}\wedge \left|T_{x}\right|\geq 2\\ ⊘ & {\mathrm{ch}}_{x}\notin T_{x}\wedge \left|T_{x}\right|\geq 2 \end{array}\right..$$

**Definition** **2.** *$V=Lock(V,p_{x},{\mathrm{ch}}_{x})$. For the arbitrary adjacent edges a and b in the path $p_{x}$ (a[3]=b[1]), the pass-through loss matrix $M=GM(v_{a[3]},{\mathrm{ch}}_{x})$ of node a[3] are updated by setting M[a[4]][a[4]]=b[2] and M[b[2]][b[2]]=a[4] when $T_{a[3]}\geq 0$, $T_{a[3]}$ is the supported wavelength channel vector of node a[3].*

**Definition** **3.** *$V=Unlock(V,p_{x},{\mathrm{ch}}_{x})$. For the arbitrary adjacent edges a and b in the path $p_{x}$ (a[3]=b[1]), the pass-through loss matrix $M=GM(v_{a[3]},{\mathrm{ch}}_{x})$ of node a[3] are updated by setting M[a[4]][a[4]]=∞ and M[b[2]][b[2]]=∞ when $T_{a[3]}\geq 0$, $T_{a[3]}$ is the supported wavelength channel vector of node a[3].*

### 4.2. The Modified Dijkstra Algorithm

**Figure 4:**
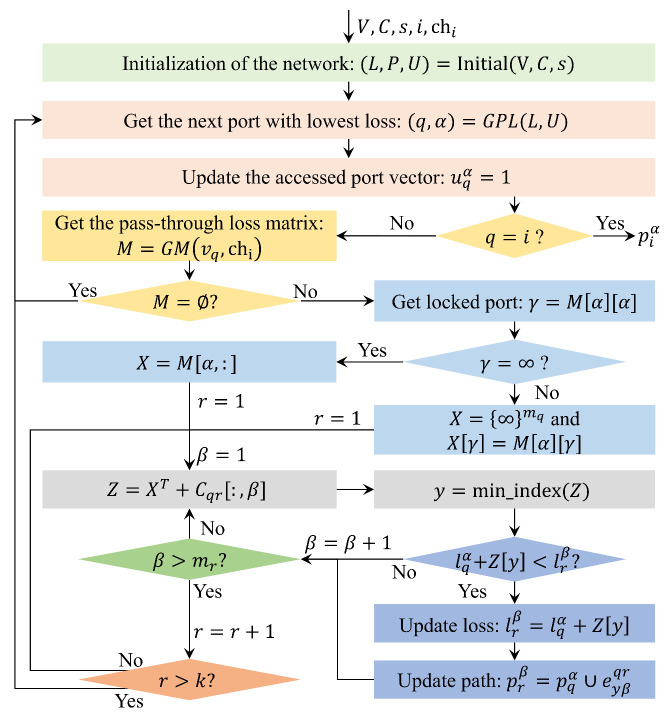
Schematic diagram of the modified Dijkstra algorithm. Initial (V,C,s) outputs $L=\{l_{\alpha }^{\beta }\}$, $P=\{p_{\alpha }^{\beta }\}$, $U=\{u_{\alpha }^{\beta }\}$, α=1,2,⋯,k and $\beta =1,2,\cdots ,m_{\alpha }$, $m_{\alpha }$ is the port number of node $v_{\alpha }$, $l_{\alpha }^{\beta }$ is the loss of the direct link between $v_{s}$ and the β-th port of $v_{\alpha }$, $p_{\alpha }^{\beta }$ is a vector composed of the edge from $v_{s}$ to the β-th port of $v_{\alpha }$, $u_{\alpha }^{\beta }$ is the binary value, $u_{\alpha }^{\beta }=1$ (0) represents the β-th port of node $v_{\alpha }$ is visited (unvisited), α=1,2,⋯,k and $\beta =1,2,\cdots ,m_{\alpha }$. In the initialization, $u_{s}^{\beta }$ is initialized to 1 and other elements of *U* are set as 0, $\beta =0,1,\cdots ,m_{s}$. $M=GM(v_{q},ch_{i})$ returns the pass-through loss matrix of $v_{q}$ for wavelength channel $ch_{i}$, if $v_{q}$ doesn’t support channel $ch_{i}$, return *∅*. C[:,β] (C[α,:]) represents the β-th column (α-th row) vector of matrix *C*. min_index(Z) represents getting the index number of the minimum value in *Z*.

The modified Dijkstra algorithm is proposed to find a lowest loss path from the entangled photon source to an arbitrary user (node *i*) with the wavelength channel ${\mathrm{ch}}_{i}$. The modified Dijkstra algorithm is shown in Figure 4, which mainly consists of eight steps.

**Step 1.** Initialize the network with (L,P,U)=Initial(V,C,s), where $L=\{l_{\alpha }^{\beta }\}$, $P=\{p_{\alpha }^{\beta }\}$, $U=\{u_{\alpha }^{\beta }\}$, α=1,2,⋯,k and $\beta =1,2,\cdots ,m_{\alpha }$. $m_{\alpha }$ is the total port number of $v_{\alpha }$, $l_{\alpha }^{\beta }$ is the link loss from $v_{s}$ to the β-th port of $v_{\alpha }$, $p_{\alpha }^{\beta }$ is a vector composed of the edge from $v_{s}$ to the β-th port of $v_{\alpha }$, $u_{\alpha }^{\beta }$ represents whether the β-th port of node $v_{\alpha }$ is visited (0 means unvisited and 1 means visited). If the β-th port of node α is not directly connected to the node *s*, $l_{\alpha }^{\beta }=\infty$ and $p_{\alpha }^{\beta }=⊘$. *U* is initialized by $U_{s}^{\beta }=1$ and other elements are 0, $\beta =1,2,\cdots ,m_{s}$.

**Step 2.** Get the next node *q* and its port α by the operation $\left(q,\alpha \right)=\mathrm{GPL}\left(L,U\right)$, which outputs node index *q* and port index α with lowest loss value in the unvisited ports. Update the accessed port vector by setting $u_{q}^{\alpha }=1$.

**Definition** **4.** *(q,α)=GPL(L,U). For ∀$\delta \in \left\{1,2,\cdots ,k\right\}$ and $\varphi \in \left\{1,2,\cdots ,m_{\alpha }\right\}$, find the node index q and its port index α, which holds $l_{q}^{\alpha }$ = $min\left\{l_{\delta }^{\varphi }+u_{\delta }^{\varphi }\times \infty \right\}$, where $L=\{l_{\delta }^{\varphi }\}$ and $U=\{u_{\delta }^{\varphi }\}$.*

**Step 3.** If q=i, output the path $p_{i}^{\alpha }$ and stop. Otherwise, get the pass-through loss matrix of node $v_{q}$ for the wavelength channel ${\mathrm{ch}}_{i}$ as $M=GM(v_{q},{\mathrm{ch}}_{i})$. if M=⊘, back to step 2.

**Step 4.** Get the locked port γ=M[α][α]. If γ=∞, get the α-th row of *M* as the loss vector *X*. Otherwise, set $X={\left\{\infty \right\}}^{m_{q}}$ and then update X[γ] as M[α][γ]. Afterwards, set r=1 and β=1.

**Step 5.** Calculate the loss from the input port α and different output ports of $v_{q}$ to the port β of $v_{r}$ by $Z=X^{T}+C_{qr}\left[:,\beta \right]$, where $C_{qr}\left[:,\beta \right]$ represents the β-th column of $C_{qr}$. Afterwards, get the row index y=min_index(Z), which holds the minimum value of *Z*.

**Step 6.** If $l_{q}^{\alpha }+Z\left[y\right]<l_{r}^{\beta }$, update the loss $l_{r}^{\beta }=l_{q}^{\alpha }+Z\left[y\right]$, update the path $p_{r}^{\beta }$ to $p_{q}^{\alpha }\cup e_{y\beta }^{qr}$, and access to next port by β=β+1. Otherwise, access to next port by β=β+1.

**Step 7.** If $\beta >m_{r}$, update the node index r=r+1 and go to step 8. Otherwise, back to step 5.

**Step 8.** If r>k, back to step 2. Otherwise, set β=1 and back to step 5.

### 4.3. Complexity Analysis

Assume the number of nodes in the network is *n* and the number of ports within each node is *m*. Our FRFS routing scheme has the time complexity of $O(m^{2}n^{2})$ and space complexity of $O(m^{2}n^{2})$.

In our FRFS routing scheme, the modified Dijkstra algorithm runs twice (finding paths from S to A and B, respectively). Thus, the FRFS routing scheme and the modified Dijkstra algorithm have the consistent complexity. The complexity analysis of the modified Dijkstra algorithm is as follows.

Time complexity: The algorithm visits the O(mn) ports with the lowest loss until the target port is found. When a port is selected, the loss and path of the other O(mn) ports are updated. Therefore, the time complexity is $O(m^{2}n^{2})$.

Space complexity: The storage is mainly consumed by variables *V*, *C*, *L*, *P* and *U*. *V* contains *n* pass-through loss matrices and each matrix has the space complexity of $O(m^{2})$. *C* contains n(n−1) link loss pass-through matrices, and the space complexity of each matrix is $O(m^{2})$. *L* is composed of mn link loss from the entangled source to all ports. *P* is composed of the mn paths from the entangled source to all ports, and each path has the complexity of O(n). *U* contains mn visited flags of all ports. Therefore, the total space complexity is $O(m^{2}n^{2})$.

## 5. Evaluation Results

In this article, We evaluate the proposed FRFS routing scheme in various entanglement distribution networks. In the evaluation, a broadband polarization-entangled photon source is used, where a 775.06-nm pump down-converts in a type-0 crystal to produce signal and idler photons with a full width at half maximum bandwidth of ≈60 nm centered at 1550.12 nm. The detailed parameters used in our evaluations are shown in Table 1.

### 5.1. Evaluation on T/W-DM Networks

**Figure 5:**
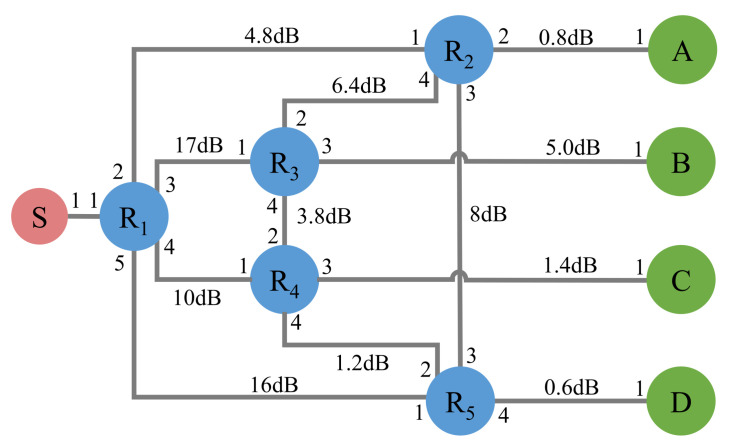
The experimental topology of T/W-DM networks. The entangled photon source (S) is connected to a WSS ($R_{1}$). They play the role of quantum server. $R_{2},R_{3},R_{4},R_{5}$ are four forwarding nodes and the numbers around the node represent the port of the device. A, B, C, and D are four users.

The FRFS routing scheme is first evaluated on the topology which originates from the SECOQC QKD network and the Tokyo QKD network and is shown in Figure 5. The entangled photon source (S) is connected to a WSS or DWDM ($R_{1}$). They play the role of quantum server, which assigns the available wavelength channel to users when they request the channel resources. $R_{2},R_{3},R_{4},R_{5}$ are four forwarding nodes and the numbers around the node represent the port of the device. A, B, C, and D are users.

The current entanglement distribution networks with dynamic topology mainly adopt the time division multiplexing (TDM) technology and wavelength division multiplexing (WDM) technology. In this article, we mainly simulate the TDM network and the TDM + WDM network by deploying different devices on $R_{1}$, $R_{2},R_{3},R_{4},R_{5}$. The detailed configurations of each device are shown in Table 2. The assigned wavelength channels for A and B are CH33 and CH35 respectively.

The paths and the link loss of user A (B) are calculated by the FRFS routing scheme under different configurations. The detailed results are shown in the Table 3. Although the networking equipment are changed, the FRFS routing scheme can find the lowest path with different loss for users. It shows that the scheme can be applied to dynamic topological networks composed of various devices. Notablely, the network in case 5 actually has only one path to establish entanglement for user A and B due to the binding of wavelength channels and ports of DWDM.

### 5.2. Evaluation with Different Sequences of Requests from Users

The FRFS scheme can also dynamically allocate the resource to users who request the resource in different orders. Based on the network of case 4 in Table 2, two different require orders for 6 pairs of users were chosen to evaluate our scheme. The detailed orders are shown in Table 4. At time $t_{1}$ of request order 1, users A and B request resources, and the FRFS scheme first searches the path from S to the former (A), and then searches the path from S to the latter (B), and the rest can be done in the same manner. The former (latter) of each pair of users is assigned to CH28-CH33 (CH40-CH35) in time order.

**Figure 6:**
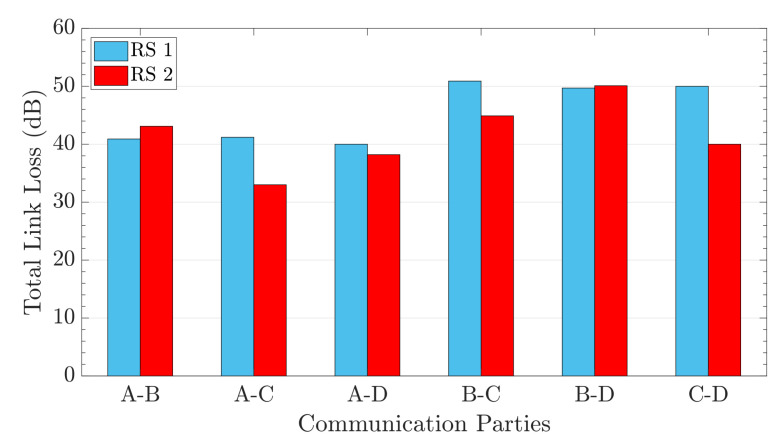
The total link loss between A and B under different orders of requests. RS 1 (2) means the request sequence 1 (2) in Table 2.

The total loss between A and B under different orders of requests is shown in Figure 6. The paths assigned to the same pair of users differ when users access the network in different order of requests. This is due to the fact that the FRFS scheme locked the links after the users request the resource.

### 5.3. Evaluation on Large-Scale and Dynamic Networks

**Figure 7:**
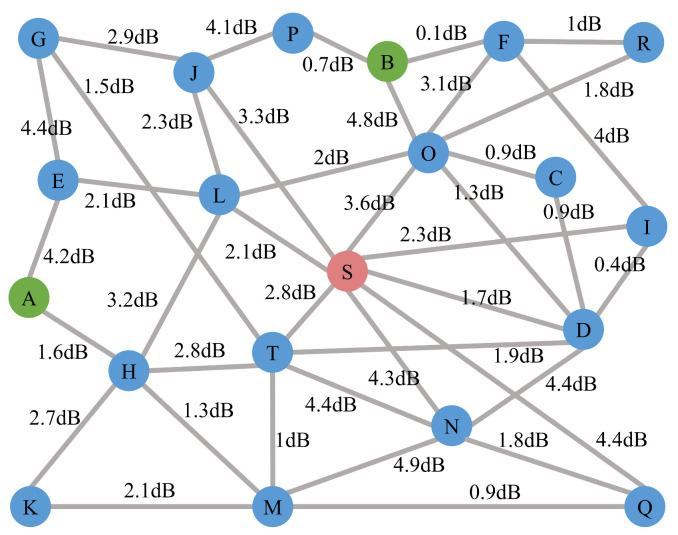
Network topology with 20 nodes and 40 edges. S is the quantum server which contains an entangled photon source and a WSS. A, B are users and other nodes are WSSs. The loss of WSS is assumed as 5 dB. The numbers on the edges indicate the link losses. The WSSs in the network can support 16 channels which contain CH26, CH27, ⋯, CH41, CH42.

We also evaluate the FRFS routing scheme on a network topology with 20 nodes and 40 edges, which is shown in Figure 7. S is the quantum server which contains an entangled photon source and a WSS. A, B are users and other nodes are WSSs. The loss of WSS is assumed as 5 dB.

As the topology may change dynamically in a large-scale network, the scheme is first evaluated on the full graph G in Figure 7 and then evaluated on the graph G with some edges cut. The assigned wavelength channels for users A and B are CH33 and CH35 respectively. The lowest paths under different topologies are calculated by the FRFS routing scheme, which is shown in Table 5. The edge loss of the path found between user A and user B is 15.3 dB when no edges of graph G are cut (without the double loss of the WSS in S and the insertion loss of intermediate nodes H, L, O). Assuming that the unit loss of the fiber is 0.2 dB/km, the fiber length between A and B can reach 76.5 km, which is longer than the point-to-point links in most metropolitan area networks [24,32,55,56]. Thus the evaluation results show that the scheme can be applied in the large-scale metropolitan area network.

## 6. Conclusions

Quantum networks enable many applications beyond the reach of classical networks, including distributed quantum computing, remote quantum clock synchronization, and distributed quantum sensing, and are already stepped into the entanglement distribution network stage. An active wavelength multiplexing entanglement routing scheme is desiderated to satisfy the dynamic connection demands of paired users in large-scale quantum networks. In this article, we first model the entanglement distribution network into a directed graph, where the nodes represent the entangled photon source, the wavelength routing devices, and the users, and the edges stand for the quantum links (fibers or free-space links) among nodes. Different to classically modeled nodes, the pass-through loss matrix among all ports within a node is described for each supported wavelength channel. Afterwards, we propose a novel first request first service (FRFS) entanglement routing scheme for quantum networks, which performs the modified Dijkstra algorithm to find the path with the lowest loss from the entangled photon source to each paired user in order. The evaluation results show that the proposed FRFS entanglement routing scheme can be applied to large-scale and dynamic topology quantum networks.

As the quantum entanglement distribution network scales up, configuring the wavelength routing network with various topologies manually is difficult for the network administrator. Using our FRFS routing scheme, the management difficulty can be significantly reduced. With the proposed weighted graph quantum network model and the modified lowest loss path searching Dijkstra algorithm, our work opens up the quality of service (QoS) entanglement routing problems for quantum networks. Various entanglement routing strategies, such as fidelity first and bandwidth first, can be further studied to provide QoS guarantees for users. Furthermore, the proposed FRFS entanglement routing scheme can be performed in quantum memory-assisted networks by replacing the QoS metric with the entanglement swapping success rate.

## Figures and Tables

**Table 1:** The parameters used in our evaluations. B is the brightness of the entanglement source. $t_{\mathrm{CC}}$ is the coincidence window and $\eta^{t_{\mathrm{CC}}}$ is the coincidence-window dependent detection efficiency. $e^{pol}$ is the individual polarization error probability. DCR is the dark count rate. $loss_{\mathrm{user}}$ is the internal optical device loss of each user.

B	$t_{\mathrm{CC}}$	$\eta^{t_{\mathrm{CC}}}$	$e^{\mathrm{pol}}$	DCR	${\mathrm{loss}}_{\mathrm{user}}$
$5\times 10^{8}$ cps	300 ps	0.761	1.81%	300 cps	3 dB

**Table 2:** Parameters of the forwarding devices in Figure 5 for different configurations. Case 1 and case 2 are TDM networks that are implemented by AOS and OS respectively. Cases 3, 4 and 5 are TDM+WDM networks and adapt the AOS to achieve TDM. The WDM in cases 3 and 4 is implemented by the WSS. In case 3, all devices support the active WDM. In case 4, partial devices (only WSS) support WDM. In case 5, the passive WDM with fixed divided channels is implemented by DWDM. (1/2in, 3/4out) indicates that ports 1 and 2 of the device are import ports, and ports 3 and 4 are output ports. The insertion losses of OS, AOS, DWDM and WSS are 0.5 dB, 1 dB, 1.5 dB and 5 dB respectively. The 5 ports WSS can support 16 channels which contains CH26, CH27, ⋯, CH41, CH42. Port 1 of the 1×4 DWDM ($R_{1}$) is the multiplexing port, and ports 2 to port 5 are demultiplexing ports that support CH32, CH33, CH35, and CH36 respectively. Port 2 of the 1×4 DWDM ($R_{2}$) is the multiplexing port, and ports 1, ports 4, ports 3 are demultiplexing ports that support CH32, CH33, CH35 respectively.

Case	Network Type	$R_{1}$	$R_{2}$	$R_{3}$	$R_{4}$	$R_{5}$
1	TDM	5 ports WSS	2×2 AOS	2×2 AOS	2×2 AOS	2×2 AOS
2	TDM	5 ports WSS	OS (1/4in, 2/3out)	OS (1/4in, 2/3out)	OS (1/2in, 3/4out)	OS (1/2in, 3/4out)
3	TDM + WDM	5 ports WSS	5 ports WSS	5 ports WSS	5 ports WSS	5 ports WSS
4	TDM + WDM	5 ports WSS	5 ports WSS	OS (1/4in, 2/3out)	2×2 AOS	2×2 AOS
5	TDM + WDM	1×4 DWDM	1×4 DWDM	2×2 AOS	2×2 AOS	2×2 AOS

**Table 3:** Paths found by the FRFS routing scheme. $l_{A}$ ($l_{B}$) is the link loss from the entangled photon source to user A (B). $loss_{u}$ is taken into account when calculating ${\mathrm{CC}}^{m}$ and $R^{s}$. $AR_{2}R_{1}SR_{1}R_{3}B$ means the path $A\leftarrow R_{2}\leftarrow R_{1}\leftarrow S\to R_{1}\to R_{3}\to B$.

Case	Path	$l_{A}$ (dB)	$l_{B}$ (dB)	${\mathrm{CC}}^{m}$ (kcps)	$R^{s}$ (cps)
1	$AR_{2}R_{1}SR_{1}R_{4}R_{3}B$	11.6	25.8	20.82	332.99
2	$AR_{2}R_{1}SR_{1}R_{3}B$	11.1	27.5	15.80	251.85
3	$AR_{2}R_{1}SR_{1}R_{2}R_{3}B$	15.6	31.2	2.39	37.63
4	$AR_{2}R_{1}SR_{1}R_{4}R_{3}B$	15.6	25.3	9.30	148.79
5	$AR_{2}R_{3}R_{1}SR_{1}R_{4}R_{3}B$	28.2	22.3	1.02	16.19

**Table 4:** The request sequences for 6 pairs of users. $t_{i}$ means the request time and $t_{i}>t_{i-1}$.

Request Sequence	$t_{1}$	$t_{2}$	$t_{3}$	$t_{4}$	$t_{5}$	$t_{6}$
1	AB	AC	AD	CD	BD	BC
2	AC	AB	CD	AD	BC	BD

**Table 5:** Paths found by the FRFS routing scheme. *G* represents the full graph in Figure 7, *G*-$e_{AH}$ represents the graph *G* without edge between node *A* and *H*.

Case	Path	$l_{A}$ (dB)	$l_{B}$ (dB)	${\mathrm{CC}}^{m}$ (kcps)	$R^{s}$ (cps)
*G*	AHLSOB	21.9	18.4	10.68	171.26
*G*-$e_{AH}$	AELSOB	23.4	18.4	7.56	121.12
*G*-$e_{OB}$	AHLSIFB	21.9	21.4	5.35	85.74
*G*-$e_{SL}$	AHTSOB	22.2	18.4	9.97	159.80

## Data Availability

The datasets and code analyzed during the current study are available from the corresponding authors on reasonable request.

## Abbreviations

The following abbreviations are used in this manuscript:

FRFS	First request first service
DWDM	Dense wavelength division multiplexer
WSS	Wavelength selective switches
BS	Beam splitter
OS	Optical switch
AOS	All-pass optical switch
TDM	Time division multiplexing
WDM	Wavelength division multiplexing
ITU	International Telecommunication Union
